# Evaluation of the Affect School as supplementary treatment of Swedish women with eating disorders: a randomized clinical trial

**DOI:** 10.1186/s40337-022-00596-9

**Published:** 2022-05-30

**Authors:** Suzanne Petersson, Kristofer Årestedt, Andreas Birgegård

**Affiliations:** 1 Faculty of Health and Life Sciences, Linnaeus University, Kalmar, Sweden; 2 Department of Rehabilitation, Kalmar Regional Council, Hus 13, plan 7, Länssjukhuset, 391 85 Kalmar, Sweden; 3 Department of Research, Region Kalmar County, Kalmar, Sweden; 4 Department of Medical Epidemiology and Biostatistics, Karolinska Institute, Stockholm, Sweden

**Keywords:** Eating disorders, Emotions, Affects, Emotion regulation, Affect School

## Abstract

**Background:**

Despite solid research there remains a large group of patients with eating disorders who do not recover. Emotion dysregulation has been shown to be a feature in the different eating disorders. A manualized group intervention developed in Sweden, the Affect School, aims to enhance emotional awareness and the ability to perceive and express emotions.

**Aim:**

This study aimed to test the hypothesis that participation in the Affect School as a complement to ordinary eating disorder treatment would enhance awareness and regulation of emotions and reduce alexithymia and cognitive eating disorder symptoms in a sample of patients with eating disorders at a Swedish specialized outpatient clinic.

**Method:**

Forty patients with various eating disorders were randomized to either participation in the Affect School as a supplement to treatment as usual (TAU), or to a TAU control group. Participants were assessed with the Eating Disorder Examination Questionnaire, the Deficits in Emotion Regulation Scale-36, and the Toronto Alexithymia Scale-20 at start, end of intervention, and at the 6- and 12-month follow-ups.

**Results:**

No significant differences were observed post-treatment but Affect School participants had improved significantly more than controls on eating disorder cognitions and behaviours and emotion dysregulation at the 6- and 12-month follow-ups and had significantly less alexithymia at the 6-month follow-up.

**Conclusion:**

Difficulties with emotion recognition and/or regulation can complicate fulfilment of personal needs and obstruct communication and relationships with others. The present study indicates that adding Affect School group sessions to regular treatment enhances emotional awareness and emotion regulation and decreases eating disorder symptoms and alexithymia.

**Plain English summary:**

Patients with eating disorder diagnoses have described problems with emotional management, for example: lower emotional awareness and difficulties in using adaptive emotional regulatory strategies compared to people without eating disorders. It has been suggested that interventions aiming at enhancing emotional awareness and acceptance would be beneficial in treatment. In the present study we explored whether adding the Affect School to regular treatment would enhance awareness and regulation of emotions and decrease eating disorder symptoms. Forty women with an eating disorder were randomly allocated to either an additional participation in a group treatment for 8 weeks or usual treatment only. The treatment contained education on different affects such as joy, fear, interest, shame, anger, disgust, and worry. The education was followed by discussions on own experiences. Participants filled in self-assessment forms that measured eating disorder symptoms, emotional recognition, and emotion regulation before the start and at the end of the group treatment, and after 6 and 12 months respectively. The results when comparing the two groups suggested that the Affect School could be an effective additional treatment. Participants in the Affect School improved their scorings but the change took time and did not show until at the 6- and 12 months follow-ups.

## Introduction

Despite decades of research on eating disorders (ED), there remains a large proportion of patients who do not recover [1, 2]. Thus, a greater understanding of the condition and more effective treatments are needed. ED are complex and emotion regulation is one of several factors that have been shown to be important to the origin and maintenance of different ED diagnoses [3]. Emotions are necessary for human survival and emotion regulation includes factors related to generation and management of affects, emotions, and other inner experiences. It can be defined as processes of recognition of, influence on, experiences, and expressions of emotions [4]. Several theoretical accounts of ED contain emotion (dys)regulation as a central component, placing emphasis on ED behaviors as maladaptive forms of escaping from negative emotion, and in turn negatively reinforcing those behaviors. For example, Haynos and Fruzzetti [5] discuss how anorexia nervosa (AN) may be based in emotional vulnerability and emotional arousal leading to ED symptoms to temporarily numb/escape distress, and invalidating responses from the social environment that hinder development of alternative, adaptive forms of emotion regulation. Both symptoms and invalidation then maintain the emotional vulnerability, forming a vicious cycle. Similarly, Integrative Cognitive-Affective Therapy (ICAT) [6] emphasizes ED symptoms as dysfunctional, temporary emotion regulation attempts, and the need for finding alternative coping strategies is a central part of the intervention. Further, the emotion dysregulation model of Gratz and Roemer [7] has been applied to both AN and bulimia nervosa, (BN), noting pervasive problems in several respects, including lack of strategies, impulse inhibition, tolerance/acceptance, and clarity in relation to distressing emotional states [8]. All of these models maintain that a relative inability to tolerate, label, and accept emotion is central to ED risk and maintenance. Such difficulties in sorting and recognizing emotions, and lack of adequate skills to handle or express feelings, lead to problems making positive long-term decisions according to valued goals, complicate relationships, and reduce opportunities for greater well-being [4, 9]. For example, restriction of food intake has been suggested to prevent experiencing negative emotion, while bingeing and purging suppress negative emotion once activated [10]. Patients with various ED diagnoses have described uncertainty regarding recognition of, as well as differentiation between, emotions, partly because the ED ‘takes over’ as the primary means of avoiding the experience of distressing emotions by behaviours and thoughts aimed towards the body (e.g., purging, restrictive eating) [11, 12]. Patients with ED have also reported less use of adaptive emotional regulation strategies compared to people in healthy control groups [3, 13].

Alexithymia is closely related to emotion regulation, a concept etymologically derived from the Greek which roughly means “lack of words for emotion”, and it has been defined as an inability to define or describe emotions, or to distinguish emotions from other bodily sensations [14]. Alexithymia has, in some studies, been shown to be over-represented among persons with ED and could be described as deficient emotion regulation [15]. It has also been shown that ED symptoms are more common in adolescents with high ratings of alexithymia [16]. Findings also suggest that alexithymia in ED could be increased or caused by the presence of anxiety and/or depression, and a full account of the causal mechanics among these constructs remains to be developed [17, 18]. However, the findings are mixed [15, 19] and it has been shown that starvation and/or affective disorders may be related to elevated alexithymia scorings in persons with restrictive anorexia nervosa but in persons with BN high ratings of alexithymia have remained after controlling for these factors [19].

Previous studies have shown that emotion dysregulation levels were reduced during ED treatment [20] and Hazzard et al. [21] showed that reductions in emotion dysregulation during Binge Eating Disorder (BED) treatment predicted improvement of eating disorder behaviour. As suggested by Monell et al. [22] and Westwood et al. [15] interventions aiming at enhancing emotional awareness and acceptance would be beneficial in treatment of the transdiagnostic spectrum of ED.

The Affect School (AS) originated from the University of Umeå in Sweden. The AS aims to increase affect awareness, the ability to perceive and express affects, in order to improve coping with stress, pain, and similar physical complaints [23]. The method is based on the affect theories developed by Tomkins, Nathanson, and Ekman and is a structured educational group intervention [24, 25]. Previous studies on the AS have shown significant reductions in stress and psychological symptoms in a sample of social workers with high levels of chronic stress [24], as well as improvements regarding alexithymia, depression, social relations, and quality of life in primary care patients [25], and improvements of depression, anxiety, and fibromyalgia in samples of women on sick leave in primary care [26]. The AS has not previously been evaluated as a method for patients with ED.

This study aimed to test the hypothesis that participation in the Affect School as a complement to ordinary eating disorder treatment would enhance awareness and regulation of emotions and reduce alexithymia and cognitive eating disorder symptoms in a sample of patients with eating disorders at a Swedish specialized outpatient clinic.

## Method

### Design

This randomized superiority trial was registered in the International Standard Randomized Controlled Trial Number (ISRCTN) registry (study ID: ISRCTN11278582). The study was approved by the Regional Ethical Review Board at Linköping, Sweden (No 2017/531-31). Self-reported outcome measures were used to assess ED, ER, and alexithymia at baseline, post-intervention, at the 6 and the 12-month follow-ups.

### Study setting

The study was conducted at a youth/adult integrated psychiatric outpatient clinic in southern Sweden, specializing in the treatment of ED. At the time of the study the clinic received approximately 150 new patients yearly. Patients with different ED were treated at the clinic and patients were diagnosed by experienced psychologists or psychotherapists who classified according to the DSM-5 by adapting suggestions from the DSM-IV-based Structured Eating Disorder Interview (SEDI) [27] to the DSM-5 criteria for ED.

### Participants and procedure

Inclusion criteria were an ED diagnosis, ongoing treatment at the clinic, ≥ 18 years of age, and Swedish speaking, while exclusion criteria were inability to participate meaningfully in AS due to BMI < 15, psychosis, suicidal tendencies, or acute starvation. The criteria were evaluated by experienced staff at the clinic and in cases where there was doubt, there was access to a medical doctor for further evaluation.

**Fig. 1:**
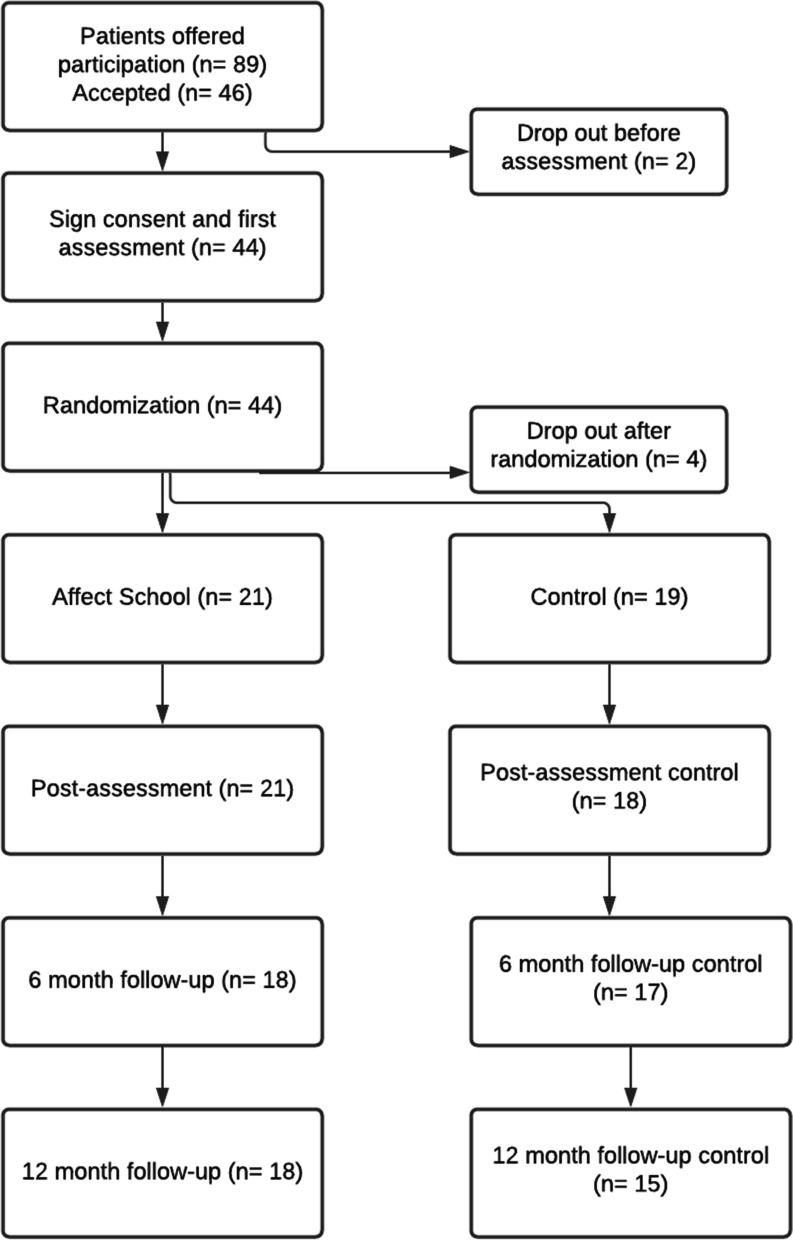
Flow chart of study process and participants

With a start in January 2018 until December 2019, 89 patients were consecutively informed about the study by staff and through posters at the reception of the clinic. After having filled in their baseline data and informed consent to participation in the study, participants were assigned by the first author to either AS or to control group (CG), using block randomization; each block included two interventions and two controls in a random order (the process is illustrated in Fig. 1). The randomization was conducted in Stata 16.1 (StataCorp LLC, College Station, TX, USA) by an independent statistician. Inclusion occurred on six occasions during this period, thus six AS groups were formed.

Forty-six patients with different ED diagnoses agreed to participate. The main reason for declining participation was not wanting to risk being randomized to the AS, since they were either negative to group participation, or lacked the time. Four participants dropped out after randomization but before the start of the AS. A total of 40 patients remained in the study, of which 21 participated in the AS while the remaining 19 were in the CG. Parallel to the intervention both groups received treatment as usual which included individual psychotherapy (CBT) and in some cases also day care and/or physiotherapy. Self-reported outcome measures were followed up at the end of the AS intervention and after 6-, and 12-months, respectively. No time-corresponding treatment with another/neutral content was offered to the CG.

### The Affect School

The affects included in the AS are joy, fear, interest/startle, shame, anger, dissmell/disgust, and worry [23]. The last session was originally devoted to education on stress, pain, and its prevention. In the present study, this theoretical section was replaced by a corresponding section on ED (after a request from the authors to Professor B-Å Armelius, the originator of the AS, personal communication 2017-09-19). The AS consisted of eight 2-h group sessions during 8 weeks [23]. The sessions were held at daytime, which affected some of the participants’ attendance at school or work. Participants who must absent from work to participate in the interventions were entitled to financial compensation from the Swedish Social Insurance Agency (Preventive sick leave). All sessions followed the same approach: initially 30–60 min’ psychoeducation about a specific affect followed by reflections and a break. Participants received handouts at the beginning of each session. After the break a discussion of the affect followed, and each group participant was encouraged to talk about an incident that had started this affect, followed by a longer reflection on the situation when the specific affect occurred, how it was experienced, how it was expressed (verbally and non-verbally), and how the participant could identify other people’s expression of the specific affect [23]. Participants did not know beforehand which affect was to be discussed at the following session, and between sessions were encouraged to reflect on the affect they had gone through during the last session [23].

The AS has been manualized, and training under supervision is required to become an AS leader. Three master’s level psychologists (all women), well educated in ED treatment and who worked at the clinic led the AS groups in the present study. The leaders worked in pairs and were AS trained.

### Data collection

The administration of outcome measures took place after consenting to participate after inclusion, and prior to randomization to either the AS or CG. At the end of the last AS session participants completed their first follow-up measures, and participants in the CG did the same in conjunction with their treatment visits. At the 6 and 12-month follow-ups participants received forms by post with accompanying, stamped, self-addressed envelopes. Non-responders were sent a second letter with self-report measures after 3 weeks.

Thirty participants filled in the outcome measures on all four occasions (16 from the AS group and 14 from the CG). One participant failed to fill in the outcome measures on the third occasion (1 CG), three participants only filled in the outcome measures on the first two occasions (1 AS, 2CG), two participants missed the third occasion (3 AS), and four participants missed the fourth occasion (2 AS, 2 CG).

### Outcome measures

For the present study, widely used self-reported outcome measures (global scores) were used to assess ED, ER, and alexithymia at baseline, post-intervention, at the 6 and the 12-month follow-ups. All outcome measures were validated Swedish versions.

*The Eating Disorder Examination Questionnaire (EDE-Q version 4.0)* [28, 29] was used to measure ED cognitions and behaviours covering the past 28 days. The EDE-Q measures concerns about shape, weight, and eating together with eating restraint. It comprises 36 items, scored 0–6, and variables are calculated as averages of included items. A higher score indicates more severe eating disorder pathology. The scale has shown high internal consistency and good test–retest reliability [29–31]. In the present study Cronbach’s *α* for the global score was 0.95.

*The Difficulties in Emotion Regulation Scale-36 (DERS-36)* [7] was used to assess emotion dysregulation. The scale measures a range of facets of responses/attitudes to emotional distress; finding it unacceptable, difficulties maintaining goal-directed behavior, difficulties managing impulses, limited access to functional emotion regulation strategies, and lack of emotional understanding and clarity as well as inattention to emotional responses and lack of emotional awareness. It comprises 36 items, scored 1–5. Variables were calculated as averages of included items and a higher score indicated greater emotional dysregulation. The scale has shown satisfactory psychometric properties [7] and has been validated in a large sample of Swedish patients with ED diagnoses. It was concluded that the total scale accounted for most of the variance in ED pathology and was recommended in ED populations [32]. In the present study Cronbach’s *α* for the global score was 0.94.

*The Toronto Alexithymia Scale-20 (TAS-20)* [33] was used to measure alexithymia. TAS-20 comprises 20 items, scored 1–5, and scores range from 20 to 100. Higher scores indicate more severe alexithymia, and a clinical cut-off score of ≥ 61 has been suggested [34]. It has shown adequate psychometric properties [33, 35]. In the present study Cronbach’s *α* for the global score was 0.85.

### Data analysis

According to an a priori sample size calculation, the required sample size was 42 participants equally distributed between the AS group and the CG. The calculation was based on an expected a medium effect size (f = 0.25), 1 − β = 0.8, α = 0.05, and ρ = 0.1. Descriptive statistics were used to describe background characteristics and study variables. Independent sample *t*-test and Person chi-square test were used to compare differences in background characteristics between the AS group and the CG. Spearman Rank-Order Correlation was used for correlation analyses between the outcome measures at baseline.

Mixed models with random intercepts were used to examine the intervention effect on the outcome variables, one model for each outcome variable, i.e., EDE-Q (ED cognitions and behaviours), DERS-36 (emotion dysregulation), and TAS-20 (alexithymia). Group (0 = CG, 1 = AS), time (dummy-coded with the baseline assessment as reference category), and the multiplicative interaction term between them, i.e., group × time, were used as explanatory variables. The fixed effects included time, group, and group × time; the random effects included the subject level. The unstructured covariance matrix was used to model the correlations among the residuals. Due to the small sample size, restricted maximum likelihood (REML) was used to fit the models [36]. Evaluated with histograms and normal probability plot of residuals and scatterplots of residuals versus predicted values respectively, no problems with non-normal distributed residuals or heteroscedasticity were detected. Statistical significance was set at *p* < 0.05. The statistical analyses were conducted in Stata 17.0 (StataCorp LLC, College Station, TX, USA) and Statistica 13.0 (StatSoft©, Tulsa, OK, USA).

## Results

### The sample

The mean age in the entire sample was 29.0 (*SD* = 9.3) and their mean BMI was 23.7 (*SD* = 6.5). Distribution of the DSM-5 eating disorder diagnoses at baseline was AN: *n* = 6 (15%), BN: *n* = 3 (7.5%), BED: *n* = 6 (15%), and Other Specified Feeding or Eating Disorder (OSFED): *n* = 25 (62.5%). Among participants with an OSFED diagnosis 20% met the criteria for atypical AN, 20% had BN of low frequency and/or limited duration, and the remaining 60% had normal weight but showed inappropriate compensatory behaviours (Table 1). Regarding the participants with AN, there were no changes in BMI from the initial assessment to the 6-month follow-up and BMI data for the 12-month follow-up was lacking for half of the study participants (three out of six participants, two from the AS and one from the CG).

**Table 1:** Characteristics of the study sample at baseline (n = 40)

	Affect school group, n = 21	Control group, n = 19	*p* value
Age, *M* (*SD*) [*Range*]	28.3 (9.6) [18–46]	29.7 (9.1) [19–51]	0.638^a^
BMI, *M* (*SD*) [*Range*]	23.5 (5.8) [16.6–38.9]	24.2 (7.4) [15.6–44.8]	0.656^a^
Eating disorder diagnoses, *n* (*%*)			0.332^b^
Anorexia nervosa	4 (20)	2 (11)	
Bulimia nervosa	0 (0)	3 (16)	
Binge eating disorder	3 (14)	3 (16)	
OSFED restrictive	2 (10)	3 (16)	
OSFED^2^ compensating	12 (57)	7 (37)	
OSFED unknown subtype	0 (0)	1 (5)	
Outcome variables, *M* (*SD*) [*Range*]			
EDE-Q	4.0 (1.2) [1.6–5.6]	3.1 (1.4) [0.6–5.5]	0.030^a^
TAS-20	63.9 (13.7) [34–87]	62.1 (9.9) [42–81]	0.650^a^
DERS-36	3.4 (0.7) [1.9–4.6]	3.2 (0.7) [1.8–4.3]	0.296^a^

BMI = Body Mass Index (weight (kg)/height (m^2^)), DERS-36 = Difficulties in Emotion Regulation Scale, EDE-Q = Eating Disorder Examination Questionnaire, OSFED = Other Specified Feeding and Eating Disorder, TAS-20 = Toronto Alexithymia Scale

^a^Independent sample t-test

^b^Fisher’s exact test

No significant differences between the AS group and the CG were found regarding age, BMI, ED diagnoses, emotion regulation, and alexithymia. However, the AS group scored significantly higher levels on the EDE-Q scale at the baseline assessment compare with the CG (*p* = 0.030). Participants in the AS scored significantly higher at the EDEQ at baseline compared to the CG. There were no significant baseline differences between the groups’ scorings on the DERS-36 or the TAS-20 (Table 1).

When comparing the AS group and the CG regarding current diagnoses at the 12-month follow-up, there were no differences χ^2^(4, *N* = 38) = 1.32, *p* = 0.857. Two participants in the CG had discontinued their treatments at the time and were thus not included in the analysis.

### Correlations between the outcome measures

The DERS correlated significantly with EDE-Q (r_s_ = 0.55, *p* < 0.001) and TAS (r_s_ = 0.65, *p* < 0.001) at baseline, while there was no significant correlation between EDE-Q and TAS (r_s_ = 0.30. *p* = 0.57).

### Intervention effects

The score distributions for the outcome variables between groups and time are presented in Table 2. Based on these descriptive statistics the same trend was shown for all outcome variables. First, the mean scores decreased over the follow-up assessments in both groups. Two exceptions were shown for the CG at follow-up 2; at this assessment EDE-Q and TAS-20 were scored higher than follow-up 1. Second, the AS group reported higher mean scores at baseline and the first follow-up while the CG reported higher mean scores at the last two follow-up assessments.

**Table 2:** Score distributions of the outcome measures at baseline and follow-up assessments

Outcomes	Groups	Measurements, *M* (*SD*)			
		Baseline	Follow-up 1	Follow-up 2	Follow-up 3
EDE-Q	AS	4.0 (1.2)	3.4 (1.4)	2.3 (1.5)	2.0 (1.3)
	CG	3.1 (1.4)	2.8 (1.6)	2.9 (1.6)	2.4 (1.7)
TAS-20	AS	63.9 (13.7)	57.0 (11.8)	46.9 (12.1)	45.6 (11.6)
	CG	61.8 (9.1)	56.8 (16.0)	58.2 (15.0)	52.3 (15.4)
DERS-36	AS	3.4 (0.7)	3.0 (0.7)	2.4 (0.7)	2.2 (0.7)
	CG	3.2 (0.7)	2.9 (0.7)	2.7 (0.8)	2.5 (0.9)

AS = Affect school, CG = Control group, DERS-36 = Difficulties in Emotion Regulation Scale, EDE-Q = Eating Disorder Examination Questionnaire, TAS-20 = Toronto Alexithymia Scale

Follow-ups: 1 = at the end of the Affect school, 2 = 6 months after ending the Affect school, 3 = 12 months after ending the Affect school

The results from the mixed models are reported in Table 3.

**Table 3:** Intervention effects based on linear mixed models with random intercepts and unstructured covariance matrix

	EDE-Q	TAS-20	DERS-36
Main effect of group			
Control group	Ref	Ref	Ref
Affect school	0.94 (0.42)*	2.02 (3.83)	0.26 (0.22)
Main effect of time			
Baseline	Ref	Ref	Ref
Follow-up 1	− 0.34 (0.30)	− 5.17 (3.14)	− 0.29 (0.12)*
Follow-up 2	− 0.37 (0.28)	− 4.26 (2.61)	− 0.46 (0.13)***
Follow-up 3	− 0.98 (0.33)**	− 11.22 (2.91) ***	− 0.68 (0.18)***
Interaction effect of time and group			
Baseline—control group	Ref	Ref	Ref
Follow-up 1—affect school	− 0.33 (0.30)	− 1.69 (4.30)	− 0.13 (0.16)
Follow-up 2—affect school	− 1.20 (0.38)**	− 11.89 (3.60)**	− 0.53 (0.18)**
Follow-up 3—affect school	− 1.08 (0.44)*	− 7.22 (3.98)	− 0.59 (0.25)*

DERS-36 = Difficulties in Emotion Regulation Scale, EDE-Q = Eating Disorder Examination Questionnaire, TAS-20 = Toronto Alexithymia Scale

Follow-up: 1 = at the end of the Affect school, 2 = 6 months after ending the Affect school, 3 = 12 months after ending the Affect school

*p < 0.05, **p < 0.01, ***p < 0.001

*EDE-Q:* The main effect of group showed that the AS group overall scored significantly higher levels on the EDE-Q compared to the CG. A main effect of time was also shown, both groups scored significantly lower levels at the follow-up 3 compared to the baseline assessment. The interaction effect showed that the AS group improved significantly more at both the second and third follow-up compared to the baseline assessment and the CG (Tables 2, 3).

*TAS-20:* No significant effect of group was shown immediately after the AS but both the AS group and the CG improved significantly at follow-up 3 compared to baseline. The interaction effect showed that the AS group improved significantly more at the second follow-up compared to the baseline assessment and the CG (Tables 2, 3).

*DERS-36:* No significant effect of group was shown immediately after the AS but both the AS group and the CG improved significantly at all follow-up assessments, compared to the baseline. The interaction effect showed that the AS group improved significantly more at both the second and third follow-up compared to the baseline assessment and the CG (Tables 2, 3).

## Discussion

The present study is one of the first to have evaluated the intervention effects of the AS in adult patients with different types of ED. The AS aims to increase awareness and management of emotional processes and, not surprisingly, participants in the AS group improved significantly more compared to the baseline assessment and to participants in the CG regarding alexithymia and emotion regulation deficiencies. However, the results partly supported the hypothesis that the intervention reduced ED symptoms; while no significant effect was seen at post-treatment, improvement was significantly greater in AS at the 6- and 12 months follow-ups. Due to initial significantly higher scorings on the EDE-Q (compared to the CG participants), participants in the AS group seemed to have greater ED difficulties which complicates the interpretation of this result.

Changes in emotion regulation, and associated changes in ED symptoms, may take time and the improvements only became apparent at the 6-month follow-up and not immediately after completion of the AS. In conjunction with the present study, we interviewed nine of the participants on their experiences of participation in the AS [37]. The interviewees felt the AS was like a piece of a puzzle, and the results of the AS were realised over time. Data from the present study showed that the interviewees were correct: differences between groups appeared at the 6- and 12-month follow-ups. And, as expected, AS participants improved significantly more regarding emotion regulation difficulties and alexithymia 6 and 12 months after the AS compared with ED patients in the CG. This is reminiscent of findings regarding emotion regulation-focused therapy to reduce self-harming behavior, where improvement in emotion regulation during treatment predicted reductions in self-harm over the 9-moth follow up, rather than being associated with improvement in self-harm during treatment [38]. This suggests that improved emotion regulation skills continued to carry an effect after treatment.

A disadvantage noticed during the recruitment of participants to the study was resistance to the group format, and several patients refused group participation. They stated that the groups disrupted school, work, and other valued activities, regardless of the time of the group. Resistance or ambivalence to group treatment [39] or treatment for ED per se is common [40, 41], which needs to be considered.

A careful interpretation of data from the present study suggests that the AS or a similar (systematic and structured) intervention could be an effective adjunctive treatment for patients with ED. Adding eight weekly 2-h group sessions would be a relatively small effort, especially if the group sizes were large enough (approximately six to eight persons per group). Since patients with ED tend to have difficulty recognizing and identifying emotions as well as regulating emotions, which in turn leads to deficient abilities in evaluation, decision making, learning enhancement, and attachment [9], they would benefit from developing this area. Further research on manualized affect- or emotion-focused programmes as ancillary treatment components, and with larger samples, is needed to draw conclusions about relationships among awareness of emotions, adequate emotion regulation, and ED symptoms.

### Limitations

This randomized study has some limitations that need to be considered. The a priori sample size calculation was conducted for repeated measure ANOVA (within-between interaction) and not mixed model. Therefore, the study may be somewhat underpowered. Previous research has found it difficult to recruit adult patients with ED to efficacy trials and adding recruitment of adolescent patients could have been considered [42]. Another research design, for example a single case design, with focus on individual responses to the AS could be argued to be more relevant than a randomized study, not least due to the small sample sizes.

Participants in this study had different ED diagnoses, which could also be considered positive since it mirrored clinical reality, but it is possible that a group of patients with restrictive AN also would have reported more difficulties [43]. Although our data did not suggest that weight restoration among AN participants was responsible for the improvement in study variables, we cannot rule out a minor such effect since we did not have BMI at follow-up for all participants. Participants in the AS group scored significantly higher on the EDE-Q compared to the CG and although their scores decreased significantly and finally ended up at the same level, this initial difference complicates the interpretation of results. It is for example possible that those who declined participation after being randomized to AS reported a lower symptom burden, which may suggest that committing time and effort to AS may be viewed as more acceptable to those with higher subjective distress.

The study took longer than expected due to slow recruitment and there was a resistance to participation in the group format. Thus, since the study was under-powered it was not possible to know if those who agreed to participate were representative of a larger sample of persons with an ED, affecting generalizability of the results. Participants in the AS received 16 more intervention hours, which should be investigated further in future studies where group meetings with other content than ED and emotion regulation questions would be addressed.

Self-report data was used, entailing risks of response bias. Further, if participants with ED have difficulties in defining and describing affects/emotions, one can question whether self-assessment scales are the right method to investigate these issues [44]. However, well established, valid, and reliable measurements were used and ensured that the study variables were measured in the same way, and an interview study was undertaken with a subsample of the AS participants which can be considered as a complement to this study [37].

## Conclusion

The present study indicates that an addition of the AS group sessions to regular treatment enhances emotional awareness and emotion regulation and decreases ED symptoms. However, effects of the intervention seem to take time. EDs are difficult to treat, and regardless of origin (such as whether the ED precedes the emotional problems or vice versa), issues related to emotion recognition and/or regulation can complicate satisfying personal needs and obstruct communication and relationships with others. Treatment of ED should thus pay attention to emotional difficulties and work with the enhancement of emotional knowledge and skills, which may reciprocally enhance the therapeutic process.

### Clinical implications

Despite solid research there remains a large group of patients with eating disorders who do not recover. Emotion dysregulation has been shown to be a feature in the different eating disorders. Treatment of ED should pay attention to emotional difficulties and work with the enhancement of emotional knowledge and skills, which may reciprocally enhance the therapeutic process. The AS could thus be an effective adjunctive treatment for patients with ED.

## Data Availability

The datasets generated during and/or analyzed during the current study are not publicly available due to patient confidentiality.

## Abbreviations

AN: Anorexia Nervosa
AS: Affect School
BN: Bulimia Nervosa
BED: Binge Eating Disorder
CG: Control group
DERS: Deficits in Emotion Regulation Scale
ED: Eating Disorder(s)
EDE-Q: Eating Disorder Examination Questionnaire
OSFED: Other Specified Feeding and Eating Disorders
TAS-20: Toronto Alexithymia Scale-20
TAU: Treatment as Usual
